# First sequence of influenza D virus identified in poultry farm bioaerosols in Sarawak, Malaysia

**DOI:** 10.1186/s40794-020-0105-9

**Published:** 2020-03-12

**Authors:** Emily S. Bailey, Jane K. Fieldhouse, Natalie A. Alarja, David D. Chen, Maria E. Kovalik, Juliana N. Zemke, Jessica Y. Choi, Laura K. Borkenhagen, Teck-Hock Toh, Jeffrey Soon Yit Lee, Kuek-Sen Chong, Gregory C. Gray

**Affiliations:** 1 Division of Infectious Diseases, Duke University School of Medicine, DUMC Box 102359, Durham, NC 27710 USA; 2 Duke Global Health Institute, Duke University, Durham, North Carolina USA; 3 Julia Jones Matthews Department of Public Health, Texas Tech University Health Sciences Center, Abilene, TX USA; 4 Clinical Research Center, Sibu Hospital, Sibu, Sarawak Malaysia; 5 Faculty of Medicine, SEGi University, Kota Damansara, Selangor Malaysia; 6 Divisional Health Office, Sibu, Sarawak Malaysia; 7 Global Health Research Center, Duke-Kunshan University, Kunshan, China; 8 Emerging Infectious Disease Program, Duke-NUS Medical School, Singapore, Singapore

**Keywords:** Influenza D virus, Influenza, Aerosol, Bioaerosol, Poultry

## Abstract

In 2018, our team collected aerosols samples from five poultry farms in Malaysia. Influenza D virus was detected in 14% of samples. One sample had an 86.3% identity score similar to NCBI accession number MH785020.1. This is the first molecular sequence of influenza D virus detected in Southeast Asia from a bioaerosol sample. Our findings indicate that further study of role of IDV in poultry is necessary.

## Background

Newly recognized as a novel species, influenza D virus (IDV) was first isolated in 2011 from a pig exhibiting influenza-like-illness (ILI) [1]. IDV has been detected in various animal species including pigs [2], cattle [3], goats and sheep [4] with the highest prevalence reported in young cattle with symptoms of bovine respiratory disease (BRD) [5]. To date, IDV has not been isolated in poultry [4]. In studies conducted in the United States, serological evidence indicates that IDV has been present in cattle populations since at least 2004 [6] and a cross-sectional study conducted in Florida detected a high prevalence (97%) of neutralizing antibodies in cattle exposed workers compared to non-exposed controls (18%) [7]. IDV transmission has also been noted in comingled cattle herd with 94% seroprevalences of IDV antibodies [8]. More recently in 2015, IDV was isolated in both pigs and cattle during a swine respiratory disease outbreak [9]. The IDV genome associated with the swine respiratory disease was closely related to the viral genome isolated in the United States in 2011. Globally, IDV has been isolated in Morocco, Togo, Benin [10], China [11], and Japan [12], but, prior to this study, has not been detected in Southeast Asia. Despite these initial detections of IDV in swine and cattle, relatively little is known about the potential zoonotic transmission of IDV to humans [1], and IDV disease has not been described in humans. Currently there is no recommended therapy or vaccine available for IDV, despite active research [13].

## Methods

From June 3 to August 3, 2018, our study team collected 28 bioaerosol samples from five poultry farms across the Sibu Division of Sarawak, Malaysia. Bioaerosol sampling was conducted using the National Institute of Occupational Safety and Health’s (NIOSH, Morgantown, West Virginia, USA) model BC 251 two-stage bioaerosol sampler calibrated at a rate of 3.5 L/min [14–16]. At the poultry farm, NIOSH samplers were fixed approximately 1 m above the ground on a stationary tripod, set-up near or inside the holding pens of the chickens or ducks for a 1.5-h period. Holding pens were either closed or covered with open-sided enclosures. After collection, samples were immediately transported back to the laboratory on ice and stored at − 80 °C until sample processing could occur.

Viral RNA extracts were analyzed with a real-time polymerase chain reaction (qPCR) for detection of influenza D virus [15] using Superscript R III Platinum One-Step qRT-PCR System with Platinum Taq DNA Polymerase (Thermo Fisher Scientific, Inc., Waltham, MA). Samples positive for influenza D virus were further amplified and sequenced using RT-PCR primers and probes [17]. Partial genome sequencing was performed by Eton Bioscience (Eton Bioscience, Inc., Raleigh, NC, USA). Sequences were then compared to the NCBI sequence database using the BLAST application of BioEdit 7.1.9 (Ibis Biosciences, Carlsband, CA, USA). Sequences were aligned and phylogenetic analysis was performed using the UPGMA method in Geneious Prime 2019.1.1 (Biomatters Inc., San Diego, CA, USA).

## Results

IDV was detected in 4 of the 28 (14.29%) samples collected from the poultry farms. One of the four IDV positive samples was successfully sequenced (1200 bp sequence) as influenza D virus. As samples were detected at low concentrations, real time PCR CT values ranged between 34 and 36, no efforts to culture these viruses were attempted.

**Fig. 1:**
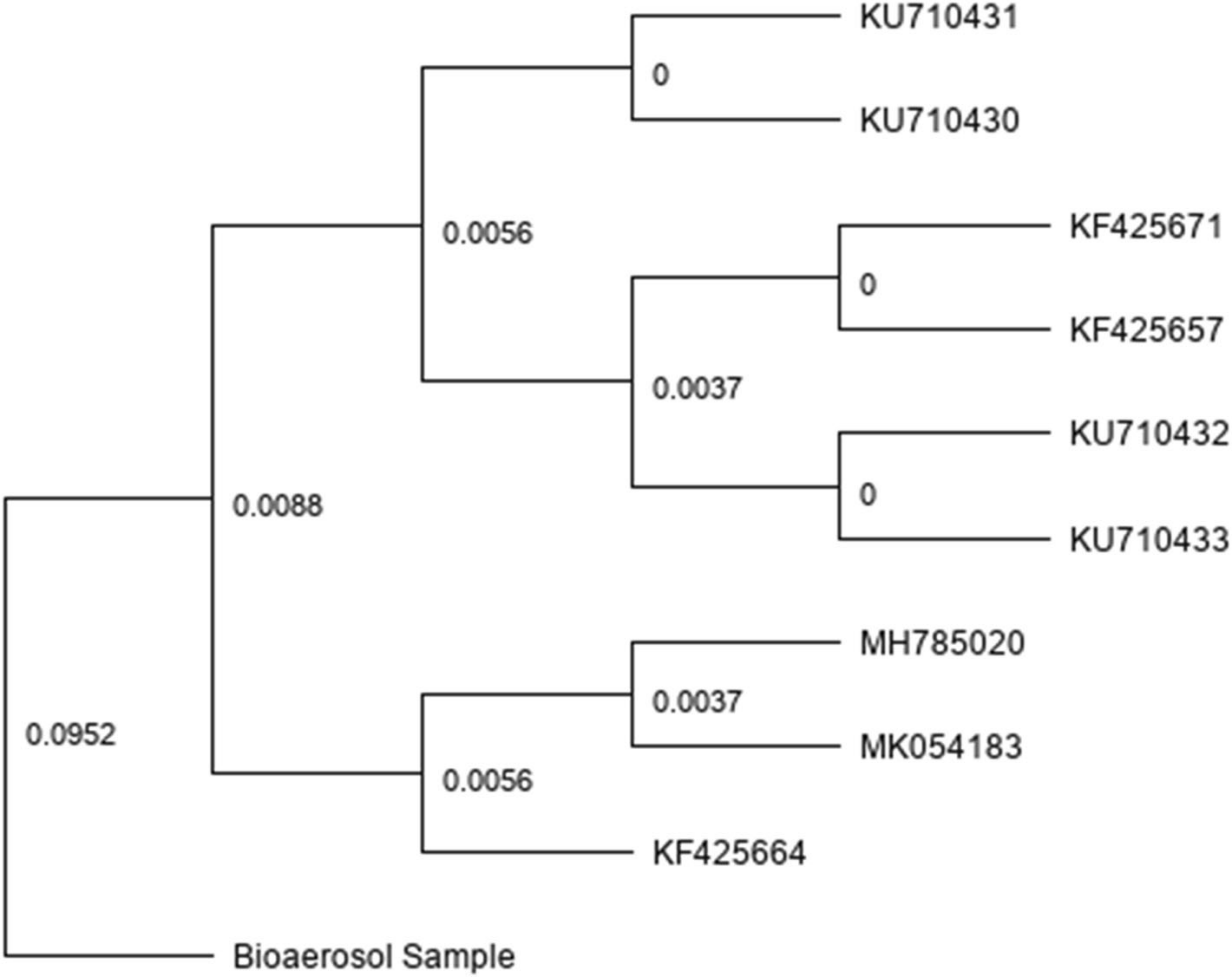
Phylogenetic analysis of 5 North American influenza D virus strains available in NCBI (accession numbers: Mexico Strain: KU710433, KU710432, KU710431, KU710430, Minnesota Strain: KF425671, KF425657, Pennsylvania Strain: MH785020, Kentucky Strain: MK054183, and Oklahoma Strain: KF425664) compared to our bioaerosol sample. The scale indicates the relative genetic distances between each isolated sequence

A phylogenetic tree of five North American influenza D virus isolate sequences currently available in GenBank and the IDV isolated in our study is illustrated in Fig. 1. This tree demonstrates that the genetic distance between strains previously isolated in North America is much less than (nearly zero genetic difference) the strain that we have isolated from our bioaerosol sample collected Southeast Asia. These results suggest that there could be different strains of IDV circulating in animal populations in Asia.

## Discussion

This study of aerosols conducted in Sarawak is the first to successfully sequence influenza D virus in an aerosol sample from a poultry farm. The results of this study as well as our previous work in an airport [16] suggest that aerosol sampling is a useful technique for respiratory virus surveillance in high traffic and areas of high human-animal interaction. Aerosol sampling has advantages in that it minimally disrupts activities in a commercial setting (abattoir or poultry farm), is simple in setup and operation, and the processing procedures for isolating the viral nucleic acid are relatively simple [16, 18].

A limitation of our study is the inability to link aerosol results with poultry stalls, chickens, or ducks. Also, the inability to link detection of aerosol positivity to presence of virus in poultry hosts, the low concentrations we detected and the lack of viral culture to access viability and infectivity are important limitations. However, the benefits of this environmental sampling approach are the early detection and screening of food animals. Another limitation is that our phylogenetic analysis was primarily focused on the comparison of this bioaerosol sample to North American strains of IDV, this could be expanded to include other reported strains. As other animals present on the farms included in this study might have also influenced the detection of IDV, additional study is needed to determine if poultry can become infected with IDV and/or transmit this virus.

Our finding that IDV is detectable in bioaerosols near poultry farms suggest that commercial food production activities may be generating infectious aerosols. Such industries may, therefore, benefit from aerosol sampling to strengthen surveillance to protect the public from respiratory viruses. This strengthened surveillance may also support public health responses to respiratory virus detection by encouraging the use protective equipment (such as respirators or masks) by at risk workers or customers.

## Data Availability

The data collected during the current study are available from the corresponding author on reasonable request.

## Abbreviations

BLAST: Basic local alignment search tool
BRD: Bovine respiratory disease
IDV: Influenza D virus
NCBI: National center for biotechnology information
NIOSH: National institute of occupational safety and health
qPCR: Real-time polymerase chain reaction
UPGMA: Unweighted pair group method with arithmetic mean
